# Characterization of cement float buoyancy in the stalked barnacle *Dosima fascicularis* (Crustacea, Cirripedia)

**DOI:** 10.1098/rsfs.2014.0060

**Published:** 2015-02-06

**Authors:** Vanessa Zheden, Alexander Kovalev, Stanislav N. Gorb, Waltraud Klepal

**Affiliations:** 1 Faculty of Life Sciences, Core Facility Cell Imaging and Ultrastructure Research, University of Vienna, Vienna, Austria; 2 Zoological Institute: Functional Morphology and Biomechanics, Kiel University, Kiel, Germany

**Keywords:** adhesive, buoyancy, foam, biological material, Arthropoda

## Abstract

*Dosima fascicularis* is the only barnacle which can drift autonomously at the water surface with a foam-like cement float. The cement secreted by the animal contains numerous gas-filled cells of different size. When several individuals share one float, their size and not their number is crucial for the production of both volume and mass of the float. The gas content within the cells of the foam gives positive static buoyancy to the whole float. The volume of the float, the gas volume and the positive static buoyancy are positively correlated. The density of the cement float without gas is greater than that of seawater. This study shows that the secreted cement consists of more than 90% water and the gas volume is on average 18.5%. Our experiments demonstrate that the intact foam-like cement float is sealed to the surrounding water.

## 1. Introduction

Barnacles (Cirripedia, Thoracica) are sessile, marine Crustacea with free-swimming larval stages. The adult barnacle is permanently attached via its proteinaceous adhesive, the so-called cement, to stationary hard materials such as rocks or to natural and man-made floating objects. Lepadomorph stalked barnacles are typical rafting organisms [1]. Their pelagic larvae colonize any floating objects such as driftwood, macroalgae, plastic or tar pellets [2–4]. They even settle on living animals, e.g. sea snakes [5,6], turtles [7], birds [8] and seals [9]. With this strategy, barnacles extend their habitat. Thus, these normally sedentary animals turn into ‘hitchhikers’ which raft through the sea [9,10]. Generally, lepadomorph barnacles secrete a thin layer of cement for attachment. One of them, *Dosima* (*Lepas*) *fascicularis* (Ellis and Solander, 1786), has evolved special adaptations. This animal produces a large amount of foam-like cement with gas-filled cells [11]. The gas is presumably CO_2_, a by-product of metabolism [12].

The cypris larva of *D. fascicularis* settles mainly on small floating objects, such as bird feathers or algae. After metamorphosis of the cyprid and as the animal grows, the amount of cement increases. It may overgrow the substratum and form a float [13,14] which gives positive static buoyancy to the animal and enables it to drift autonomously just at the water surface [1]. The cement is produced by cement glands in the upper part of the stalk, transported together with the gas through the cement canal system and then secreted through pores on the stalk base [12].

The phenomenon of positive static buoyancy is also known in other marine organisms, such as snails, cnidarians and brown algae. These also produce their own floating devices to enable them to drift through the water. The snails of the family Janthinidae secrete a float of bubbles of mucus [15,16]. With this float, they drift and hunt in the neuston in contrast to the related benthic species. Neustonic cnidarians such as *Velella velella* and *Physalia physalis* have one gas-filled bladder which acts as a sail, allowing them to float on the water surface [17,18]. Brown algae such as *Fucus vesiculosus* have gas-filled floats, called pneumatocysts, which give positive static buoyancy to the blades [19,20]. Floating closer to the water surface, these algae are exposed to more sunlight, which improves their photosynthetic activity.

Most studies on such biological floats deal with the ecology of drifting or rafting organisms [1,10,21], but to the best of our knowledge nothing is known about positive static buoyant properties of these structures. After having dealt with some mechanical properties [22], the aim of this study was to investigate the unusual function of the cement of *D. fascicularis* with special emphasis on its positive static buoyancy, its gas and water content as well as its volume–weight correlation.

## 2. Material and methods

**Figure 1.**
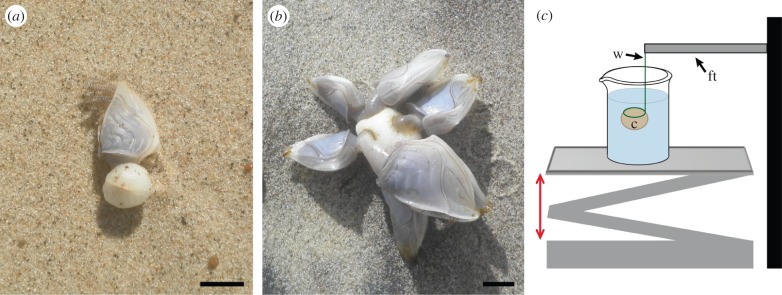
(*a*) Single *Dosima fascicularis* washed ashore. (*b*) Several animals shared one cement float. (*a*,*b*) Scale bar, 1 cm. (*c*) Experimental set-up for measuring the positive static buoyant force. The cement ball (c) floating in seawater was pushed under the water surface with a thin wire frame (w) attached to a force transducer (ft). The double-headed arrow indicates the movement of the platform.

Individuals of the stalked barnacle *D. fascicularis,* which had been washed ashore, were collected on the northwest coast of Denmark. The animals occurred either individually (figure 1*a*), in groups on the same cement float (figure 1*b*) or attached to small floating objects (e.g. feathers). For our investigations, we used individuals from colonies of two to seven animals, attached to one float, with a capitulum length between 2 and 3 cm. For the experiments, only the cement floats detached from the animals were used. Prior to the analyses the separated cement was stored in seawater with a 2% antibiotic antimycotic solution (Sigma-Aldrich, Vienna, Austria).

### 2.1. Microscopy

Razor blade sections through whole cement floats were photographed with a Lumix DMC-GH1 camera (Panasonic, Hamburg, Germany) mounted on a MZ3000 stereo microscope (Micros Austria, St Veit/Glan, Austria). For scanning electron microscopy, cross sections of the float were fixed in 2.5% glutaraldehyde in 0.1 mol l^−1^ sodium cacodylate buffer with 10% (w/v) sucrose at pH 7.3 for 2 h and rinsed in distilled water. After air-drying, the cement samples were coated with gold by an Agar B7340 sputter coater (Agar Scientific Ltd, Stansted, UK) and examined in a Philips XL 30 scanning electron microscope (FEI/Philips, Eindhoven, The Netherlands) at 15 kV.

### 2.2. Mass, volume and water content

For measuring the water content of the cement, the floats were wiped with tissues to remove any excess water. Only traces of salts from the seawater may have been left on the surface of the floats. Ten cement floats were weighed and their volumes were determined by water displacement. Two floats were then air-dried for 2 days; in addition, three floats were dried in the oven at 65°C overnight. Afterwards the dried cement floats were weighed again. The water content was calculated by subtracting the dry mass from the wet mass.

### 2.3. Buoyant force and gas volume

Ten cement floats were placed one after the other in a 100 ml beaker filled with seawater. Each float was totally submerged to a depth of about 1 cm below the water surface by a thin wire frame attached to a force transducer (FORT-100) using a motorized micromanipulator (DC3001R; both from World Precision Instruments, Sarasota, FL, USA) (figure 1*c*). The floats were kept underwater for about 20 s and their positive buoyant force was measured. The accuracy of the positive buoyant force measurement was about 0.07 mN. The data were collected with the software AcqKnowledge v. 3.7.0 (Biopac Systems, Inc., Goleta, CA, USA). Afterwards, volume, mass and positive buoyant force of the cement floats were determined. The volume of the gas *V*_g_ inside the cement float was calculated using the following equation:
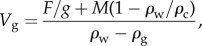
where *F* is the positive buoyant force, *g* is the gravitational acceleration (9.81 m s^−2^) and *M* is the mass of the dried cement float. The density of the seawater *ρ*_w_ (1018.65 kg m^−3^) is calculated as mass divided by volume. Because of the irregular internal structure of the cement float (due to the greatly differing size of the gas-filled cells) and the resulting uncertainty in determining the density of the cement, we used the standard value *ρ*_c_ = 1350 kg m^−3^ for the density of proteins in our calculations [23,24]. *ρ*_g_ is the density of CO_2_ (1.977 kg m^−3^) within the cells [12].

### 2.4. Reduced pressure experiments

In a vacuum desiccator, four cement floats were placed in a beaker filled with seawater. Two of them were exposed to a pressure of 700 mbar and two others to 30 mbar. A total of 30 mbar pressure was reached in about 20 min evacuation time. During pressure reduction, the floats were observed to detect any structural changes and any escaping gas bubbles. After the desired pressure was reached, the floats were kept at this pressure for 5 min. The positive buoyant force of the floats at atmospheric pressure (996 mbar) was measured before and after the reduced pressure treatment.

### 2.5. Statistical analyses

The regression calculations were made via a random bootstrap approach [25,26] (with 10 000 iterations) by the program routine MUREG from a software package of computer intensive statistics [27]. Graphs and histograms for all experiments were created in Sigma Plot v. 11.0 (Systat Software Inc., Bangalore, India).

## 3. Results

### 3.1. Cement morphology

**Figure 2.**
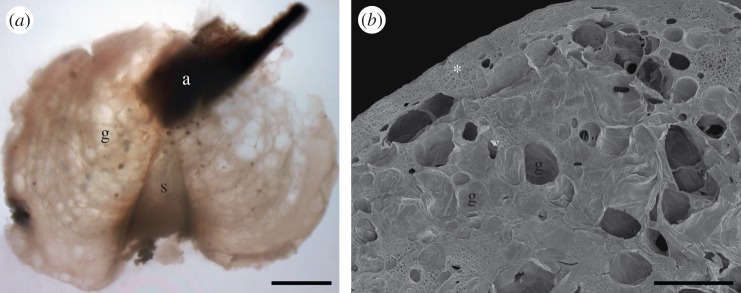
(*a*) Stereo microscopic image of a razor blade section through a cement float (partly torn). The cement formed concentric layers around the stalk (s) and the alga (a). Gas-filled cells (g) were obvious in the cement matrix. Scale bar, 2 mm. (*b*) Scanning electron micrograph of a cross section of a cement float. Large gas-filled cells (g) were mainly in the inner region of the float, whereas small, round cells (*) prevailed in the outer region forming a kind of rind. Scale bar, 1 mm.

The cement was secreted in concentric layers around the stalk and the attachment site (figure 2*a*). Large gas-filled cells of irregular shape were mainly found in the inner region of the cement float. Small, round cells dominated in a kind of rind near the surface of the float (figure 2*b*).

### 3.2. Mass, volume and water content

**Figure 3.**
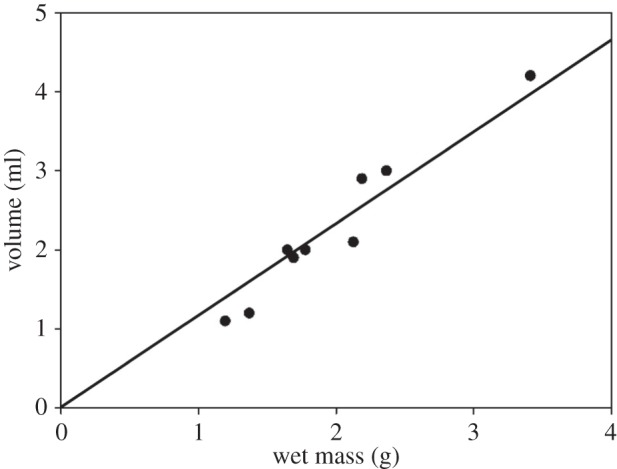
Correlation between the volume of the cement float and its wet mass. The data were fitted by random bootstrap for linear regression: *y* = 1.16*x*, *R*^2^ = 0.92, *p* < 0.001; *R*^2^ = coefficient of determination.

There was a significant correlation between the volume of the cement float of *D. fascicularis* and its wet mass (*R*^2^ = 0.92, *p* < 0.001). The greater the volume, the heavier the float (figure 3). The number of animals attached to the same cement float did not influence mass and volume of the float. Only the size of the animals was crucial. For example, one float with four animals attached was heavier and bigger than the floats with seven animals (table 1).

**Table 1.** Summary of the physical values measured in 10 floats to which a varying number of animals were attached.

float number	animals attached	wet mass of the float (g)	volume of the float (ml)	volume of the gas (ml)	buoyant force (mN)
1	2	1.19	1.1	0.17	1.53
2	2	1.78	2.0	0.35	3.14
3	2	2.12	2.1	0.13	0.97
4	4	1.19	1.1	0.29	2.69
5	4	1.64	2.0	0.54	5.06
6	4	3.41	4.2	0.97	9.05
7	5	2.37	3.0	0.33	2.9
8	7	1.37	1.2	0.27	2.45
9	7	1.69	1.9	0.27	2.33
10	7	2.19	2.9	0.62	5.84

**Figure 4.**
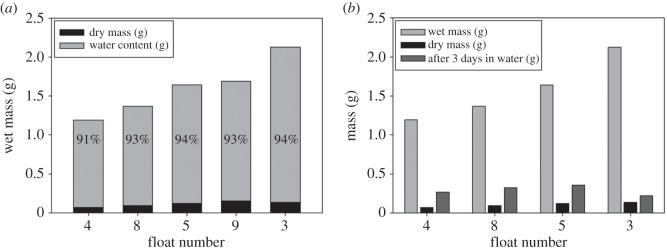
(*a*) The bar plot shows the wet mass of five floats, which consisted of the dry portion (black) and the water portion (grey) of the cement. Floats 4 and 8 were air-dried; floats 3, 5 and 9 were oven-dried. (*b*) The bar plot shows the wet mass (light grey), the dry mass (black) and the wet mass of the dried floats kept in seawater again for 3 days (dark grey). After 3 days in seawater, some dried floats took up more than 20% of water but they did not return to their original mass.

The water content of the cement floats was over 90% (w/w), no matter whether they had been air-dried or oven-dried (figure 4*a*). When the dried cement was immersed in seawater again, it still floated. Within a few minutes, the cement float became heavier because of water uptake. After 3 days in water, the air-dried cement floats were more than three times heavier than the dry mass, but they did not return to the original wet mass. Interestingly, the oven-dried floats did not take up as much water as the air-dried ones (figure 4*b*).

### 3.3. Buoyant force and gas volume

**Figure 5.**
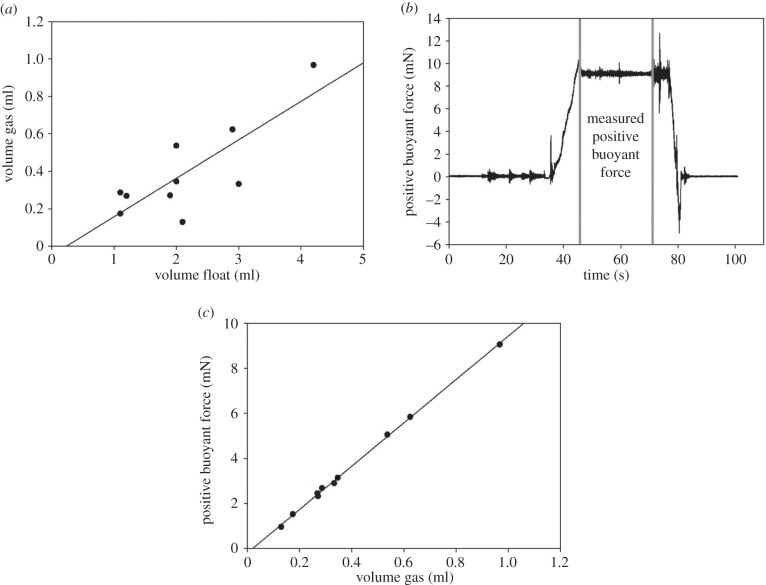
(*a*) Correlation between the volume of the gas inside the cement and the volume of the float. The data were fitted by random bootstrap for linear regression: *y* =−0.05 + 0.2*x*, *R*^2^ = 0.65, *p* = 0.018. (*b*) Graph of a typical positive buoyant force measurement. The positive buoyant force of a cement float was defined as an average force in the area between the two grey lines (in this case 9.05 mN). (*c*) Correlation between the positive buoyant force and the volume of the gas inside the float: *y* =−0.19 + 9.6*x*, *R*^2^ = 0.99, *p* < 0.001.

The volume of the gas was linearly dependent on the volume of the float (*R*^2^ = 0.65, *p* = 0.018) (figure 5*a*). In essence, the bigger the float, the higher the gas volume. The gas volume fraction in a float was on average 18.5% ± 6.7 (*n* = 10). A typical graph of the measured positive buoyant force is shown in figure 5*b*. In this figure, the force required to hold the cement float underwater was 9.05 mN. The volume of the gas inside the float and the positive buoyant force were significantly correlated (*R*^2^ = 0.99, *p* < 0.001) (figure 5*c*). Clearly, the higher the gas content in the float, the more positive static buoyancy it had.

### 3.4. Reduced pressure experiments

**Figure 6.**
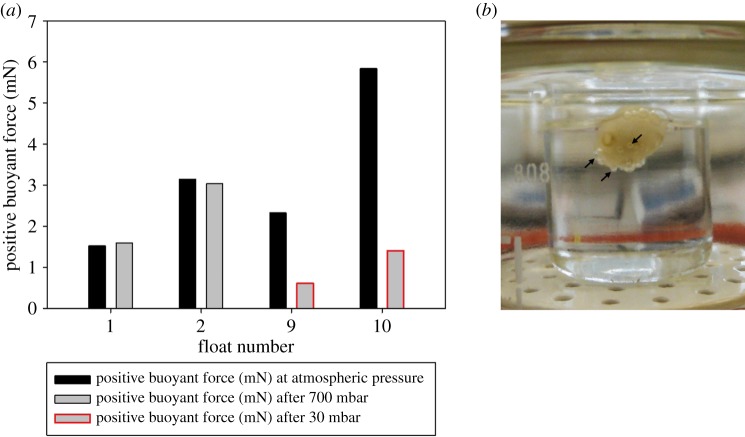
(*a*) Differences between the positive buoyant force of the cement floats at atmospheric pressure (black) and after the exposure to reduced pressure: 700 mbar (grey) and 30 mbar (grey with red frame). (*b*) Cement float in a beaker filled with seawater in a vacuum desiccator below 400 mbar. Gas bubbles leaked out of the burst cement cells (arrows).

For the pressure experiments, two cement floats, kept in seawater, were exposed to 700 mbar, and two others were exposed to 30 mbar ambient pressure. There was almost no difference between the positive buoyant force measured before and after treatment with 700 mbar underpressure (figure 6*a*: floats 1 and 2). During continuing pressure reduction to 30 mbar gas bubbles were observed leaking out at a pressure of about 400 mbar. They accumulated at the surface of the float (figure 6*b*, arrows), which started to crack. Below 250 mbar most cells in the cement had burst, water began to fill the voids and the positive buoyant force decreased. Even after exposure of floats to 30 mbar underpressure, the cement floats still had some positive buoyancy (measured at ambient pressure), but in comparison with the floats exposed to 700 mbar underpressure, the positive buoyant force was much lower (figure 6*a*: floats 3 and 4). A few hours after exposure to 30 mbar underpressure the floats sank. Owing to water uptake the mass of the floats had increased. After 5 days in seawater, the cement was more than 25% heavier than measured immediately after the 30 mbar underpressure experiment.

## 4. Discussion

The stalked barnacle *D. fascicularis* is unique among the barnacles because it is able to secrete its own float. It can even form colonies attached to one central float [28]. Interestingly, the volume and mass of the float are primarily correlated with the size of the attached animals and not with their number. The colonies investigated in this study contained barnacles of similar size. This was presumably the result of several cypris larvae having settled on the same substratum during the same reproductive period, as was also previously suggested by Ryan & Branch [28]. The increasing cement of the adult animals eventually formed one joint float.

### 4.1. Water content

The original wet cement of *D. fascicularis* had a water content of more than 90% (see also [29]). After air-drying, the cement took up more water than when it was oven-dried. A possible explanation is that the protein structure of the cement changes as a result of irreversible protein denaturation during heating up to 65°C. Another reason for the difference in water uptake after the process of drying could be that air-drying is slower and thus gentler than oven-drying. The air-dried material did not shrink so much and therefore water had easier and faster access into the cement again. The reason for dried floats not returning to their original mass after rehydration might be that the immersion time was not long enough.

### 4.2. Buoyancy

Rafting is a typical feature of lepadomorph barnacles. *Lepas anatifera* and *L. testudinata* attach with a thin layer of cement to large and highly buoyant objects such as kelp or plastic [30]. By contrast, *D. fascicularis* normally settles on small floating objects, which are often overgrown by the cement of the adult. By developing a foam-like cement full of gas-filled cells in accordance with the growth of the animal *D. fascicularis* maintains positive static buoyancy of the cement throughout its lifetime [2,28].

For the estimation of the cement density, we used the value 1350 kg m^−3^ of protein density [23,24]. According to this value, the density of the proteinaceous cement was higher than that of seawater (1018.65 kg m^−3^). Consequently, without gas-filled cells the cement would sink. In principle, the greater the volume of the float the bigger the gas volume, but the gas content of the floats varied between *D. fascicularis* colonies (table 1). As expected, the floats with less gas content had lower positive static buoyancy than floats with higher gas content. The gas volume and thus the positive static buoyancy of the float could also depend on the object to which the float was attached. The use of floating objects, such as feathers, for attachment causes positive buoyancy in itself. In this case, the volume of the cement does not need to be large to compensate for the weight of the attached animals.

### 4.3. Reduced pressure experiments

To test whether the cement float is an open or closed porous system we exposed the floats, which are drifting at the water surface, to underpressure. In a closed porous system an increase in underpressure should eventually result in bursting of the cement cells. The subsequent gas escape would be visualized by gas bubbles released at the interface between the water and the float. In an open porous system, we assume that gas exchange would take place through the part of the float outside the water.

In our experiments at atmospheric pressure and at underpressure of 700 mbar, the positive buoyancy of the float was unaffected and even continuous evacuation down to 400 mbar had no visible consequences for the float. At an ambient pressure below 400 mbar, the cement burst and gas bubbles were seen at the cement–water interface. Through the cracks in the cement, water could penetrate the voids and the float began to sink. The fact that the floats sank due to structural failure of the cement gave further evidence that, although the intact cement float was porous, it was sealed to the surrounding water.

## 5. Conclusion

All lepadomorph barnacles raft by attaching with their cement to natural or artificial drifting substrata. In *D. fascicularis*, the cement has a double function: adhesion and floating independent of any drifting substratum. The prerequisite for the use of the *D. fascicularis* cement as a float is its positive static buoyancy depending on the balance between the volume of the float and the volume of the gas within the cement cells. The mass and the volume of the float correspond to the size of the attached animals and not to their number. The water content of the float is more than 90% and the gas volume is on average 18.5%. Although the float is porous, it is crucial that it is sealed to the surrounding water by a kind of rind which also gives mechanical stability and forms a barrier to the environment.

The cement of *D. fascicularis* is an interesting example of how the adhesive material can change its function due to a slight modification of the structure. The use of the cement as a float at the water surface allows the essentially sessile animal secondary mobility. By this means, *Dosima* can extend its habitat and occupy new ecological niches.
